# The Impact of Mast Cells on the Anatomy, Cellular Communication, and Molecular Immune Network of Lymph Nodes

**DOI:** 10.1007/s12016-025-09050-5

**Published:** 2025-04-02

**Authors:** Daniel Elieh-Ali-Komi, Marcus Maurer, Frank Siebenhaar

**Affiliations:** 1 Institute of Allergology, Charité – Freie Universität Berlin and Humboldt-Universität Zu Berlin, Berlin, Germany; 2 Fraunhofer Institute for Translational Medicine and Pharmacology ITMP, Immunology and Allergology, Berlin, Germany

**Keywords:** Mast cells, Lymph nodes, Medulla, Cortex, Follicle, Germinal center, Lymphangiogenesis, Hodgkin’s disease

## Abstract

Lymph nodes (LNs) are ovoid-shape capsulated structures interposed along the lymphatic vessels. Owing to their unique architecture, LNs place immune cell types in distinct compartments allowing effective contact of antigens to them. Their efficient function results in the concentration of antigens and bridging of antigen-presenting cells like DCs and B cells and cells of adaptive immunity (circulating B and T lymphocytes remaining in LNs to monitor antigens) to coordinate efficient immune responses. In a healthy LN, B cells are primarily clustered in lymphoid follicles, whereas T cells are organized in the deeper paracortex region. Mast cells (MCs) are among the immune cells; their normal presence or pathologic infiltration has been reported in LNs. MCs enter LNs through afferent lymphatic vessels and can be found in all compartments, ranging from subcapsular sinus to the deepest sections of medullary sinus; however, they are commonly found in the T cell zone and medullary sinus but rarely in follicles. In pathologies with LN involvement and solid tumors, features like MC accumulation and the anatomical region of accumulation within LNs differ based on the type of tumor and the organ. Moreover, MC accumulation in LNs may influence the trafficking of other cell types and immune responses. MCs out of LNs can facilitate the migration of DCs into LN, which is crucial for orchestrating immune responses, especially in vaccination; moreover, MCs play a role in the induction of peripheral tolerance. MC-released mediators including TNF from tissue-resident MCs and tryptase from LN-MCs mediate hyperplasia and extension of LN vasculature, respectively. MCs support lymphangiogenesis by releasing VEGF-C and VEGF-D in vivo. Further research on the role of MCs in LNs is anticipated due to the development of pharmaceuticals that impact MC survival or inhibit their activation. In this review, we summarize the current literature regarding the outcomes of MC presence in LNs with a focus on the MC-mediated immune responses in two categories: direct cell-to-cell and mediator-based interactions.

## Introduction

The “information superhighway” title is given to lymphatic vasculature as the lymph carries the majority of inflammation sources to lymph nodes (LNs), where the lymph contents are continuously and meticulously monitored [1]. The human lymphatic system exhibits greater anatomical complexity compared to mice, with 500–800 LNs in humans versus only 22 in female BALB/cAnNCrl mice [2–5]. Secondary lymphoid organs consist of the spleen (the largest), LNs, Peyer’s patches (PPs), mucosal tissues such as nasal-associated lymphoid tissue (NALT), and tonsils [6–8]. LNs are mainly scattered in clusters in the neck, axilla, thorax, and abdomen [2] and functionally facilitate the exposure of a comparatively low number of lymphocytes to a large number of invading pathogens and biomolecules responsible for the initiation of inflammation. This outstanding capability of LNs is due to their unique histoarchitecture, which provides the opportunity for a variety of antigen-presenting cells (APCs) to effectively activate B and T cells [2]. Lymphocytes capable of recognizing the presented processed antigens by MHC-II molecules on APCs undergo clonal expansion to produce new lymphocytes and plasma cells expressing the same epitope-recognizing receptor [9, 10].

**Fig. 1:**
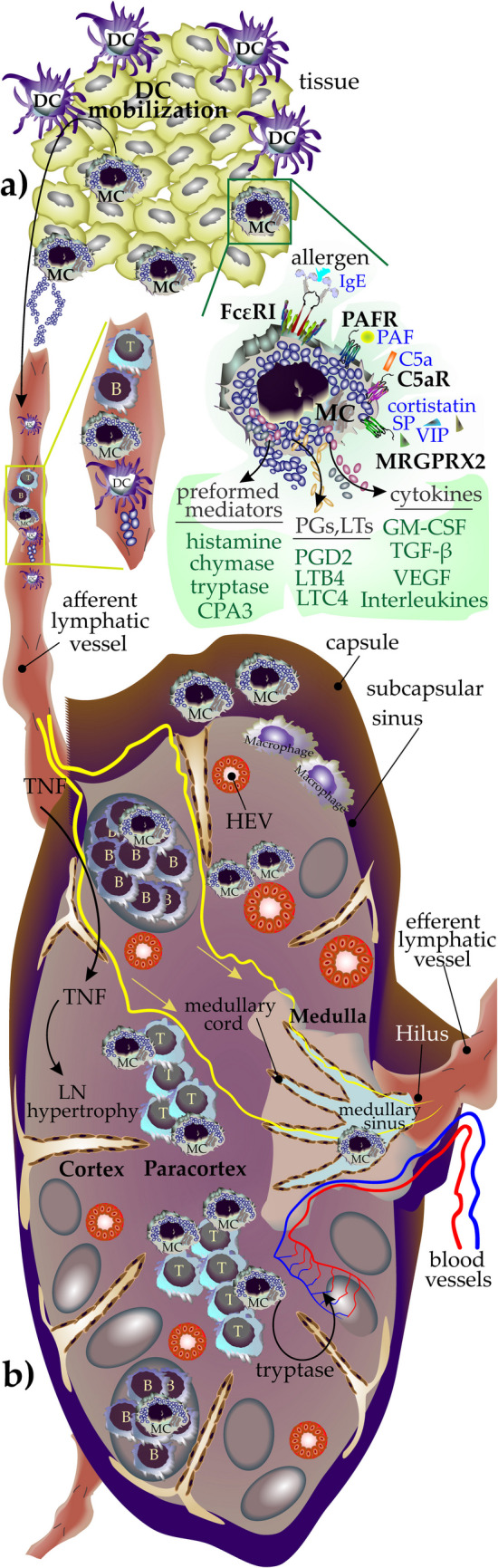
Main pathways of MC activation and their accumulation sites within LNs. **a** MCs become activated in tissues like skin upon engagement of their activation receptors like FcεRI, MRGPRX2, PAFR, and C5a (receptors are shown in black and their corresponding ligands in blue) and, in return, release three categories of mediators including (1) preformed mediators such as histamine, chymase, tryptase, and carboxypeptidase A3 (CPA3), (2) de novo synthesized mediators mainly mediators derived from arachidonic acids like prostaglandins (PG) and leukotrienes (LT), (3) a variety of cytokines like IL-10, IL-8, IL-5, IL-3, IL-1, GM-CSF, TGF-β, and VEGF. MCs release mediators that induce the mobilization of DCs from tissues into LNs to present the engulfed antigens to lymphocytes and to orchestrate adaptive immune responses. **b** MCs enter the LNs through afferent lymphatics. They can be found (or infiltrate) in different compartments of LNs (shown in the figure) like the T cell zone and medullary sinus and to a lesser extent in the follicle. Additionally, MCs release tryptase that induces neovascularization which is a crucial step in hypertrophy of LNs after inflammation or infection. MC-released TNF also plays a role in the hypertrophy of LNs

Among the immune cells of which presence in LNs has been documented are the mast cells (MCs), which are determined by the expression of CD117 (c-KIT, the receptor for stem cell factor (SCF)) and FcεRI (the high-affinity receptor for Immunoglobulin E (IgE)) [11–13]. These multifunctional effector cells originate from hematopoietic MC progenitors (MCps), showing CD34 and CD117 positivity in the bone marrow [14]. MCs become activated once their activating surface receptors are engaged with their corresponding ligands (for example, FcεRI: IgE, Mas-related G protein-coupled receptor X2 (MRGPRX2): substance P (SP), vasoactive intestinal peptide (VIP), cortistatin, platelet-activating factor receptor (PAFR): PAF, and C5aR: C5a) [15–17]. Activation and degranulation of MCs results in the release of three main classes of mediators: (a) preformed mediators including histamine, tumor necrosis factor (TNF), and the protease content mainly tryptase, and chymase; (b) de novo-produced components enzymatically synthesized from arachidonic acid such as prostaglandin (PG)D2, leukotriene (LT)B4 and LTD4; and (c) a spectrum of cytokines including IL-1β, IL-3, and transforming growth factor (TGF)-β [18–20] (Fig. 1a).

Mice with c-kit mutants have developed into an effective tool for determining and measuring the roles of MCs in a variety of biological responses in vivo. In Kit^W/W−v^ mice, the c-kit mutations affect melanogenesis and cause sterility, anemia, and significantly lower tissue MC counts. Kit^W−sh/W−sh^ mice, which have the W-sash (W^sh^) inversion mutation, lack MCs but do not exhibit anemia or sterility [21]. Additionally, several other MC-deficient mice have been introduced which bear mutations not related to c-kit. Since these newly developed strains have no c-kit mutations, the mutation does not affect other cell types and biological systems. For instance, Mcpt5-Cre; R-DTA mice are generated by crossing MC protease (Mcpt)5-Cre transgenic mice with R-DTA^fl/fl^ mice. In these mice, diphtheria toxin alpha chain (DTA) is produced only in Cre-expressing cells. Moreover, in Cpa3^Cre/+^—“Cre-Master” mice, Cre expression is induced under the control of the Cpa3 promoter while deleting 28 nucleotides of the first exon of Cpa3, which encodes for carboxypeptidase A3 (CPA3) [22].

MCs have been reported in various LN compartments, and their distribution and density (thereafter, we will mention it as mast cell density (MCD)) depend on the type of LN or the pathology. The findings of human and mouse research indicate that MCps enter LNs, produce cytokines during maturation, and along with other MCs recruited to LNs interact with other immune cells and shape the LN microenvironment [23]. Both MCs residing in LNs or tissues such as skin can be activated and release mediators that influence the biology of LNs or mediate the crosstalk between MCs and LN- structural or immune cells (Fig. 1b).

MC granules, rich in negatively charged heparin, have been reported to release from tissue MCs after horseradish peroxidase (HRP) injection. Granules enter LNs via lymphatics where they attach to the surface of reticular cells and macrophages which are negatively charged. Fibronectin aids this binding, potentially preventing electrostatic repulsion between granules and cell membranes. The granules are shown to be taken up by these cells, enter the cells, and exert their biofunctions [24].

Cottier, Turk, and Sobin at the University of Berne, Switzerland, first noticed the need to report MCs in pathologies with LN involvement in 1973. They created sheets listing cells to be reported during LN microscopic examination [25]. Over time, as more was learned about MCs’ role in diseases, reporting LN-MCs became more important. In this review, we cover different aspects of MC-mediated alterations on LN cellularity, immune cell recruitment, and interaction between MCs with a variety of immune cells and stromal cells of LN.

## Histoarchitecture, Cell Distribution, and Physiology of Lymph Node

**Fig. 2:**
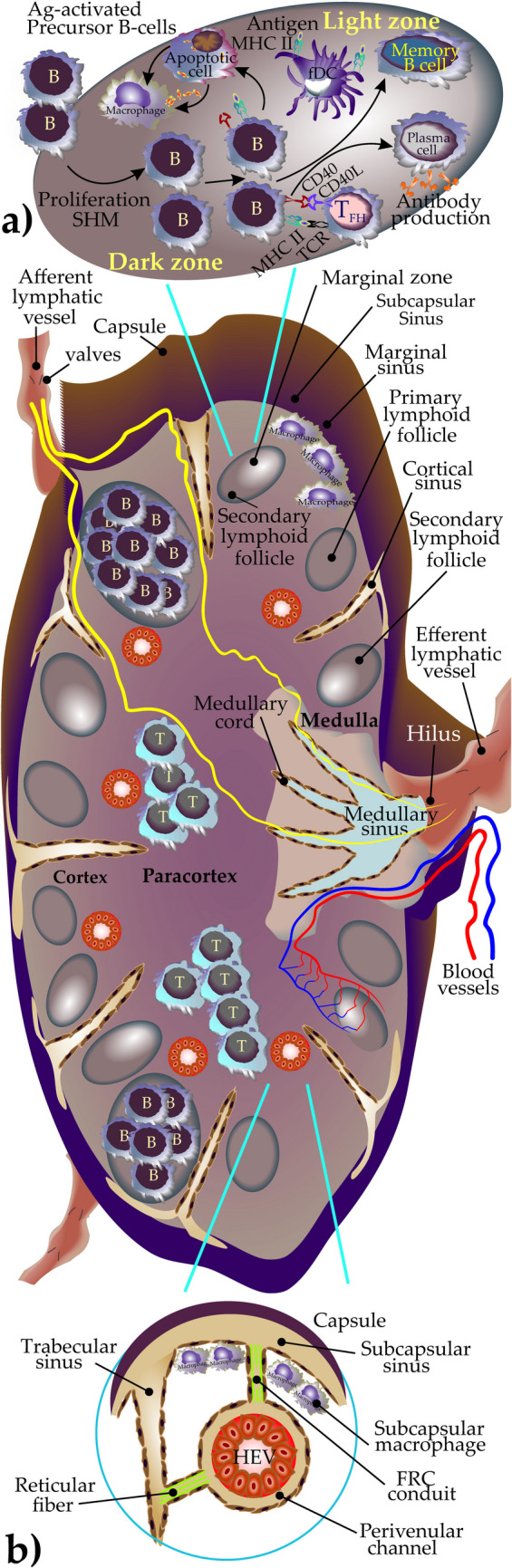
Structure, compartment, and flow of lymph and blood supply of a typical lymph node. **a** The B cell zones in lymph nodes are typically found in the LN cortex and are situated in the primary follicles. Germinal centers (GCs) are composed of the dark (DZ) and light zone (LZ). In DZ, activated B cells differentiate and undergo clonal expansion. During proliferation, the process of somatic hypermutation (SHM) is completed. B cells in LZ retrieve Ag presented by follicular dendritic cells (fDCs). The positively selected cells in LZ develop into plasma cells or memory-B cells. T cell zones in LNs are predominantly located in the paracortex. **b** The cortex, paracortex, medulla, and sinuses are the main compartments of a LN. Lymph is drained in LN through afferent lymphatics into the subcapsular sinus and traverses LN via the intermediary and medullary sinuses before draining into the efferent lymphatic vessel. Lymphocytes are drained from the circulation into LNs through the high endothelial venules (HEVs) which are surrounded by concentric layers of pericytes called fibroblastic reticular cells (FRCs)

LN is an ovoid-shaped lymphoid structure; its convex surface receives afferent lymphatics through which lymphocytes enter the structure. The presence of a valve in afferent and efferent lymphatics contributes to the stream of lymph in one direction and prevents retrograde flow. The concave surface (Hilum) places the vein, artery, and efferent lymphatics [26]. LN is a highly organized structure built up of four fundamental compartments: the cortex, paracortex, medulla, and sinuses. The capsule is made up of dense connective tissue stretching inward (trabeculae) and forms partitions [3]. Beneath the capsule, in the subcapsular sinus, a layer of macrophages (subcapsular sinus macrophages) directly exposed to the afferent lymph acts as the first line of defense against lymph-carried(borne) antigens [27]. The cortex lies within the periphery of the LN just under the capsule. It consists of the outer and inner cortex (paracortex). The outer cortex histologically contains an abundant number of B cells organized into follicles (B zone). Primary follicles are made up of small mature B cells that are naive to antigens, whereas secondary follicles develop when primary follicles respond to antigen stimulation. The presence of follicular macrophages in the area contributes to the clearance of apoptotic B cells (and their intracellular self-antigens), therefore avoiding secondary necrosis and autoimmune activation [28]. These macrophages recognize apoptotic B cells and their fragments and later mature into tingible body macrophages (TBMs) [28] Occasionally, germinal centers (GCs) are developed during T cell-dependent immune responses [29]. GCs are composed of two areas, the dark and light zones (DZ and LZ, respectively). In DZ, activated B cells differentiate into centroblasts and undergo clonal expansion. During proliferation, the process of somatic hypermutation (SHM) takes place. B cells in LZ (centrocytes) retrieve Ag presented by follicular dendritic cells (fDCs). The positively selected cells in LZ will be chosen to develop into plasma cells or memory-B cells [30] (Fig. 2a).

Deeply stretching into the cortex, the paracortex is the T-cell-rich region (T zone) located between B-cell follicles. It consists of small mature T cells and large transformed immunoblasts (T cells or B cells), interdigitating dendritic cells (iDCs), plasmacytoid dendritic cells (pDCs), and high endothelial venules (HEVs) [31]. HEVs get their name because of their distinctive “high” cuboidal endothelial cells. Intravascular lymphocytes use HEVs as passages to enter LNs [1].

The HEV is surrounded by concentric layers of pericytes called fibroblastic reticular cells (FRCs) [32]. FRCs physically support LN architecture and vasculature and produce extracellular matrix (ECM) components. After the onset of inflammation, the expanding ability of FRCs enables the LNs to become larger (swelling) to place the infiltrated and newly generated immune cells and extended vascular system [33].

Additionally, FRCs play a role in the homeostasis of T lymphocyte subsets by producing IL-7, which is indispensable for naïve and memory T cell survival. IL-7 produced by peripheral LN FRCs regulates T-cell homeostasis. It has been reported that peripheral LN FRC-derived IL-7 is of limited importance for the local and systemic survival of naïve T cells. However, the maintenance of central memory T cells critically relies on steady-state levels of FRC-derived IL-7 [34].

Interestingly, FRCs can attract CCR7-expressing cells (DCs, T, and B cells) into LNs by releasing chemokines CCL19 and CCL21 [35]. CXC-chemokine ligand 13 (CXCL13) + BRCs (a type of FRCs) networks may play a role in establishing soluble and immobilized CXCL13 gradients which mediate B cell homing and follicular scanning [36, 37]. Close to the LN hilum, the medulla occupies the core part of the LN. Medullary cords contain abundant plasma cells, macrophages, and memory T cells [31].

The sinuses are endothelium-lined lymph-carrying vessels that flow through LNs. Lymph carried in the afferent lymphatic vessel enters the node through the capsule into the subcapsular sinus (marginal sinus) and traverses LNs via the intermediary and medullary sinuses before draining into the efferent lymphatic vessel [3]. The portion of the sinuses surrounding the trabeculae is called trabecular sinuses (cortical sinuses). The extension of these sinuses in the medulla shapes medullary sinuses, which later form efferent lymphatics. A variety of cells mainly histiocytes, lymphocytes, plasma cells, and granulocytes are found in lymphatic sinuses [3, 27, 31]. (Fig. 2b).

## Mast Cell Migration to Lymph Nodes

Investigation of local MCD in tissues with inflammation in a number of diseases showed that MC number significantly increases in various anatomic sites, which is linked to clinical manifestations and the prognosis. Two good examples of these are local accumulation of MCs in the bone marrow of patients with Waldenström’s macroglobulinemia (higher MCD correlates with poorer prognosis and lower overall survival) [38] and multiple myeloma (MCD correlates with matrix metalloproteinase-9 (MMP-9, a zinc-containing endopeptidase responsible for the degradation of ECM proteins[39, 40]), receptor activator of nuclear factor κB ligand (RANKL), and osteolysis) [41]. But the question is how MCs accumulate locally in inflamed tissues. The increase in migration of MCps mediated by alteration of chemokine network or released mediators may be a widely accepted mechanism [42]; however, migration of mature MCs to other tissues has also been reported in mice system [43].

Interestingly, the recirculation of hematopoietic stem and progenitor cells (HSPCs) from extramedullary tissues back to BM through lymph circulation has been reported. In an experiment, the fluorescently tagged, HSPC-enriched thoracic duct cells were intravenously injected into recipient mice and detected in the recipients’ BM and spleen after 24 h [44].

Another level of MC migration has been documented in LNs. In a study, Wang et al. sensitized C3H/HeN and BALB/c mice with dinitrofluorobenzene (DNFB) in acetone/olive oil onto the shaved abdomen or footpads, and for controls, they applied the vehicle control (acetone/olive oil). The mice were sacrificed on different days, and the skin and LNs were used for histological tests such as toluidine blue staining, macrophage inflammatory protein-1a (MIP-1a) or MIP-1b, and in situ hybridization for MIP-1b and MIP-1a mRNA expression. Their results showed that DNFB sensitization induced the MIP-1a and MIP-1b mRNA (and protein) expression in the skin (mostly in keratinocytes and fibroblasts, respectively). MCs were activated after DNFB sensitization. Moreover, anti-MIP-1a Ab inhibited DNFB-induced MC degranulation (confirmed by toluidine blue staining). They found that DNFB sensitization reduced MCD in the skin and caused their expansion in LNs (baseline in control: 85.364.4 MCs/mm^2^ vs second DNFB exposure 198.766.5 MCs/mm^2^, *P* < 0.01). The results also showed that draining LNs had a 2.5-fold increase in weight and a threefold increase in the numbers of nucleated cells. BMMC labeled with either PKH-26 (red fluorescence) or PKH-2 (green fluorescence) were injected into left or right footpads, respectively, to track their migration from the skin to LNs. After 48 h, the left footpads were treated with DNFB and the right footpads with control vehicle. Investigation of fluorescent-labeled MCs after the second exposure showed that only an occasional green-fluorescent MCs signal was detected in the LNs from the right leg, suggesting a minimum rate of MC migration from the skin to LNs after exposure to control vehicle. However, a strong red fluorescent signal was detected in the subcapsular region of the popliteal LNs from the left leg in DNFB-exposed legs. The lack of red fluorescent MCs observed in the contralateral right popliteal LNs was used to rule out the possibility of migration via circulation instead of the lymphatic system [45].

The current literature supports the idea that MCD in LNs may increase in several pathologies and affect the prognosis of the disease. MCs likely migrate through afferent lymphatic vessels into LNs; however, further investigation is needed to clarify the mechanism of migration, especially in humans.

## Mast Cell-Mediated Induction of Peripheral Tolerance

MCs participate in immunoregulation by (1) expressing immunomodulatory receptors, such as OX40L and 4–1BB (CD137), and programmed death-ligand (PD-L1), (2) production of anti-inflammatory mediators (mainly IL-10), and (3) interaction with Tregs [46–48]. The involvement of MCs in autoimmune renal disease and necrotizing glomerulonephritis was studied by an Australian research group using wild-type (WT) and Kit^W−sh/W−sh^ mice. They induced experimental anti-myeloperoxidase (MPO) glomerulonephritis and triggered it by injecting an anti-glomerular basement membrane antibody to activate the recruited neutrophils, degranulate them in the site and to deposit MPO in the glomerular capillary bed and recruit MPO-specific CD4 + cells. Their findings showed that MPO-immunized Kit^W−sh/W−sh^ mice had enhanced focal glomerular necrosis, fibrin deposition, glomerular CD4 + T cells, macrophages, and proteinuria when compared to control C57BL/6 mice. Investigation of local LNs showed that the percentage of foxp3 + CD4 + Tregs and IL-10 production was reduced in Kit^W−sh/W−sh^ mice. Reconstitution of MC population in MC-deficient mice could attenuate glomerular injury, suggesting that MCs play a key role in autoimmunity. Additionally, the percentage of CD4 + foxp3 + cells in LNs was also elevated after MC reconstitution [49]. Although the results highlighted the role of MCs in immunoregulation observed in this study, still there was an unmet question; “Do MCs alter the immune responses reported in LNs remotely or do they become recruited from the MPO-immunization site to LNs?” The comparison of LN-MCD between WT-MPO immunized and WT-MPO non-immunized mice helped to provide an answer. The histologic investigation of LNs of these two mice groups on the sixth day revealed a four-fold increase in MC number in the immunized group, indicating that MCs have recruited to LNs. Moreover, using confocal immunofluorescence microscopy, they visualized MCs and Tregs in close proximity in LN. Another remaining question was “How do MCs influence the immunosuppression of Tregs when they collocate in the LNs?”. Coculture of MCs either from WT and IL-10-deficient mice with Tregs and effector T cells in vitro (with cell ratio 1:1:32, respectively) provided the mechanism of MC immunomodulation. WT MCs could notably enhance MPO-specific Treg suppression indicated by a 40% reduction in T effector cell proliferation and their production of proinflammatory cytokines IFNγ, IL-17A, and IL-6, while the IL-10^−/−^ MCs showed no impact, suggesting that MC-produced IL-10 plays a key role in the process [49].

Ultraviolet radiation (UV), especially UVB wavelengths (280–320 nm), possesses immunosuppressant activity mediated by inhibition of antigen presentation [50, 51], induction of immunosuppressive cytokines release, and apoptosis of leukocytes [18, 52, 53]. MCs have already been reported to have a role in UVB-induced immunosuppression. In line with this, in a paper with a well-organized methodology, several strains of mice including C57BL/ 6 J, DBA/2, and BALB/c, representing different dermal MCD, the correlation between MCD and the rate of UVB-induced immunosuppression were studied. The results showed that there was a linear relationship between MCD (C57BL/6 J > DBA/2 mice (*P* < 0.05) > BALB/c mice (*P* < 0.05)) and the log of the dose of UVB causing 50% suppression of systemic contact hypersensitivity responses. Additionally, reconstitution of the skin of MC-depleted *W *^*f*^*/W *^*f*^ mice with WT-BMMCs restored the mice’s susceptibility to UVB irradiation [54].

Byrne et al. tried to find a mechanism delineating the role of MCs in UVB-induced immunosuppression and tracked this concept into the LNs using three types of mice: WT C57BL/6, MC deficient Kit^W−sh^/^W−sh^, and green fluorescent protein (GFP) + mice (C57BL/6-Tg (UBC-GFP). They exposed mice to UV radiation (290 to 400 nm), which significantly suppressed contact hypersensitivity. The exposure affected the cellularity (thus size) of LNs. MC-deficient mice, unlike WT mice, were resistant to these effects, but injection of MCs from WT mice to their dorsal skin restored their sensitivity. The dynamics of MCD in exposed skin demonstrated an increase 6 h post-exposure, returning to normal range at the 24-h time point. Exactly at the same time, the LN-MCD doubled (*p* = 0.004). LN-MCs in irradiated and unirradiated mice were found in different compartments, in which in unirradiated mice, they were found in the subcapsular sinus and medulla regions while in irradiated counterparts were often found close to CD19 + B cells. Grafting GFP + skin onto MC-deficient mice and monitoring the MC migration pattern enabled the researchers to distinguish between circulating MC progenitors (MCPs) and MCs migrated from the skin into LNs. The results showed that GFP + cells were recruited only to LNs of the dorsal skin in irradiated mice. Flow cytometric investigation of the LN cells showed that the majority of GFP + migrated cells were CD11c- (to distinguish from DCs), CD117 + FcεR1α + suggesting that MCs after exposure to UVB migrated from the skin to LNs. But what was the molecular mechanism behind this process? They reported that MC migration to the B cell zone of the draining LNs was due to UVB exposure effect on the upregulation of CXCL12 expression in LN-B cells. CXCL12 ligand recruits CXCR4-expressing MCs from the skin to LNs. CXCR4 antagonist AMD3100 could abrogate UV-induced immune suppression [55].

Jet propulsion-8 (JP-8) is a jet fuel that already has been reported to suppress contact hypersensitivity (CHS), delayed-type hypersensitivity, and T-cell proliferation in mice. Limon-Flores et al. studied the JP-8 immunosuppression properties and linked it to MCs in C57BL/6 wild-type mice. They found an increase in skin MCD and then LN-MCD in mice after applying JP-8 on the skin of mice. Application of JP-8 to the dorsal skin of Kit^Wsh/Wsh^ could not suppress CHS. However, reconstitution of BMMCs from WT mice could restore JP-8-induced immune suppression in MC-deficient mice. Additionally, BMMCs from PGE2-deficient mice could not restore the effect, suggesting a role for PGD2. Similar to the previous study, CXCL12:CXCR4 axis was responsible for recruiting the MCs into LNs [56].

## Mast Cell Accumulation and Activation in Lymph Nodes

### MC Accumulation in Humans LNs

MCs are normally not an abundant cell type in LNs. However, MCD increases once LNs are affected by an oncologic pathology such as lymphoma (in which clusters of MCs can be observed scattering in the neoplastic LN sections), metastasis, or invasion of pathogens [57].

Recent findings reported that MCs’ presence in human tumor-draining LNs may be linked to the skewness and polarization of other immune cells. The extent of tumor-infiltrating lymphocytes (TILs) infiltration into tumors has created the concepts of hot and cold tumors, in which hot tumors, unlike cold tumors, are characterized by abundant TILs within their microenvironment. In one study, it has been reported that IL4 + MCs are linked to preferred Th2 polarization in tumor-draining LNs by enriching within the sinus and T cell zones of tumor-draining LNs associated with cold triple-negative breast cancer tumors. In these tumors, an increased Th2/Th1 ratio within cold tumors was observed, and co-staining IL4 and CD117 showed that IL4 + MCs were significantly (*P* = 0.01) higher in tumor-draining LNs in cold tumors compared to hot tumors [58].

MCs have been reported to accumulate in T cell zones but not B follicles in LNs of patients with lung cancer [59] and other pathologies; however, their localization is largely dependent on the type of pathology or involved infection. Table 1 summarizes more findings with statistical calculations based on current knowledge.

**Table 1:** MC distribution in different anatomic sites of LNs in pathologies

Type of the studied lymph node	MC density and anatomic distribution in local LNs	Ref
Data obtained from human research		
Mediastinal LNs from lung cancer patients	• MCD in tumor-free LNs was higher than metastatic LNs • In tumor-free LNs, MCs were primarily found in the T-cell area • Subcapsular sinus, paracortical areas, medullary cords, and medullary sinus • Rarely observed in the B cell zone • In metastatic LNs, MCs were primarily seen around tumor deposits and occasionally were seen within the metastatic foci	[59]
Axillary LNs of patients with breast cancer	• Survivors and nonsurvivors had a similar pattern of MC distribution • Rarely can be seen in the B zone (0.03%). MCs are found primarily in the T zone • The highest MCD was found in the medullary sinuses (47.58%) • The afferent lymphatic route was found to have a high MCD	[60]
Axillary LNs of patients with breast cancer	• MCs were found in relatively low numbers in sinus histiocytosis (8 ± 6 MCs/0.1 mm^2^) (sinuses are tightly filled with uniform oval histiocytes) • MCs were most frequently reported in the mixed sinus reaction (20 ± 16 MCs/0.1 mm^2^) (The histiocytes are less closely packed in the sinuses, cells commonly have vacuolated cytoplasm, rich in lymphocytes) • Very low numbers of MCs were found in sinus catarrh (2 ± 1 MCs/0.1 mm^2^). (The sinuses are dilated and have lower cellular content)	[61]
Axillary LNs of patients with breast cancer	• In tumor-free LNs the mean MC count was 35.75/sq mm, however, in the metastatic nodes the MC count was inversely proportional to the amount of metastatic tumor • The MC number was lower (25.64/sq mm) when the metastasis developed in other enlarged axillary LNs • MCs were mostly distributed around the tumor deposits	[62]
Axillary LNs from patients with invasive ductal breast cancer and paracolic LNs from patients with adenocarcinoma of the large intestine	• In axillary tumor free- LNs, sinus, and medulla had significantly higher numbers of MCs than LNs with metastasis • In axillary LNs, MCs had notably higher density in sinus (10/0.55 mm^2^) when compared to that of paracolic LNs (5/0.55 mm^2^), (*P* < 0.001) • The frequency of sinus MC did not differ significantly between the nodal-negative and the nodal-positive groups	[63]
LNs from the tongue, buccal mucosa, and oral cavity from patients with Oral squamous cell carcinoma (with or without LN metastasis)	• There were more MCs in LNs of the nonmetastatic group (5.81 ± 3.41) than the metastatic group (4.17 ± 2.89), (*P* = 0.034) • A weak positive correlation between the mean number of MCs in tumor and mean number of MCs in LNs was reported (correlation: 0.237 for the metastatic group and 0.091 for the nonmetastatic group)	[64]
Data obtained from mice research		
Popliteal LNs after injection of sheep RBCs or syngeneic kidney cells	• MCs accumulated after 5 h in particular in subcapsular sinuses or under them and were rarely found in the cortex or medulla • MCD returned to normal values after approximately 24 h	[65]
Mesenteric LNs of Osborne-Mendel rats infected with *Nippostrongylus brasiliensis*	• In mesenteric LNs of control rats, nearly half of the MCs were located in the capsule and a further 36% in the hilus, while a very low percentage was observed in the medulla • MCs accumulate in LNs after infection occurred in two peaks one on the 5th day and restricted to the entrance of LNs, the other characterized by a high number of MC accumulation from day 15 onward through the afferent lymphatics, and MC migration from the cortical to the medullary region	[66]

### MC Accumulation in Rodent LNs

In rodents, MC accumulation in LNs depends on the LN location, the LN compartment, the age of animal, and the normal function of other lymphatic tissues. One comprehensive study covering all these aspects was conducted by Sainte-Marie and Peng using a rat model. For this study, they grouped the rats into normal euthymic Sprague–Dawley, germfree euthymic CD, gnotobiotic euthymic Sprague–Dawley, and athymic rats and studied LN-MCD in cervical, brachial, and mesenteric LNs of rats aging between 2 and 12 months. As per their findings, aging was often accompanied by an increase in MCD, in particular, in athymic rats. In compartments of a few LNs of some aged athymic and euthymic rats, the MCD was greatly increased in the extrafollicular zone overlying medulla directly, which was accompanied by a marked MC degranulation and fibrosis in the zone. As for the distribution of MCs in LN compartments, MCs could hardly be found in the subcapsular sinus, except in an occasional node of an aged rat. In young euthymic rats, MCs were largely located in medullary sinuses. In the young euthymic rats, much fewer MCs were observed in the cortex than in the medullary sinuses [67].

Interestingly, MC accumulation may be influenced by the type of challenging antigens. In line with this, in a mouse model, 24-h post-injection of protein antigens including bovine serum albumin (BSA), porcine γ globulin, and ferritin, MCs accumulation into draining-LN interfollicular cortex or medulla is speeded up [68], highlighting their participation in immunologic responses. The observed accumulation is time, dose-dependent, and limited to specific anatomic sites of LNs, suggesting that their piling up is strictly tuned [68]; though, injection of non-immunogenic (or those with low capacity of induction immune responses) substances such as sheep RBCs or syngeneic kidney cells has also been reported to alter the mice LN-MCD as well [65].

MCs express a variety of pattern recognition receptors (PRRs) including toll-like receptors (TLRs), enabling them to recognize invading pathogens, their structural components, or released materials [69, 70]. The results of Dabak et al. delineated the reaction of MCs to the presence of bacterial components, the dynamics of MC recruitment into LNs, and collaboration to generate immune responses in Wistar rats receiving *Salmonella paratyphi* B-H antigen solution subcutaneously through the cervical region. First, let us take a look at MC numbers in different LN compartments in control rats: subcapsular area (10.15 ± 0.9/2mm^2^), the paracortical area (7.85 ± 0.7), and in the medullary sinuses (25.0 ± 0.8). Six hours post-antigen injection, no secondary follicle was observed in the stimulated group and no marked change in MC distribution was reported. On the second day, a substantial number of MCs were seen (115.0 ± 7.6/2 mm^2^) (*P* < 0.001) in the subcapsular area, 35.4 ± 1.8 (*P* < 0.001) in the paracortical area, and 45.8 ± 1.7 (*P* < 0.001) in the medullary sinuses. On the third day, the pattern of distribution changed, and a lower number of MCs was observed in the mentioned sites of LNs; instead, their numbers increased around the blood vessels (MC populations were also observed near the HEVs). Monitoring MCs on the fourth day showed that some degranulated MCs were present in the medullary sinuses and around blood vessels. Moreover, MCD increased in the subcapsular, paracortical, and medullary sinuses when compared to controls. Finally, on the eighth day, an increased number of secondary follicles were observed. Assessment of MCD showed that there were 8.0 ± 0.9 /2 mm^2^ MCs in the subcapsular area, 39.4 ± 3.2 (*P* < 0.001) in the paracortical area, 131.0 ± 13.2 (*P* < 0.001) in the medullary sinuses, and 90.2 ± 4.7 in the hilus [71].

It should be noted that MC accumulation in LN may be induced by physical factors too. Prieto et al. reported an increased rate of MC infiltration in the Wistar rats intestinal and mesenteric LNs with partial portal vein ligation (triple stenosing ligation), suggesting MCs have a role in the portal prehepatic hypertensive enteropathy [72].

The immunogenicity capacity of antigens may affect the normal dynamic of MCD in LNs. When antigens such as sheep erythrocytes (with immunogenicity capacity due to their heterologous nature and source) are injected in mice, the dynamic of LN-MCD regularly follows a pattern in which it primarily increases but within a few hours MCD gradually decreases, and in 24–48 h, the number of MC decreases below the normal values [65]. The immunobiological background of this pattern is yet to be deciphered, but a group of researchers has already reported important aspects of it. In their study, Balb/c x DBA/2W F1 were generated and injected with different types of cells either allogenic (splenocytes of strain 129) or semi-syngeneic (leukemic L1210 cells, passaged on DBA/2W mice, splenocytes or thymocytes of DBA/2W) to check their immunogenicity capacity on the dynamic of LN-MCD. The results showed that injection of normal syngeneic cells did not cause any decrease in LN-MCD; however, a decline in the number of MCs after injection of allogeneic lymphocytes or semi-syngeneic leukemic cells occurred along with a significant LN enlargement, suggesting that the observed lower LN-MCD was not because of decreasing in LN cellularity [73]. Table 1 represents the MC distribution in LN compartments in pathologies in humans and mice.

According to the following table, MCD and MC distribution in LNs are influenced by several parameters including (a) type of pathology, tumor, and its anatomical site; (b) MCD may vary in LNs of patients with metastatic and nonmetastatic tumors; (c) the anatomical distribution of MCs may differ in LNs of mice and humans and such difference could be due to structural differences observed in both LN types; (d) the type of antigen (such as parasites) may determine the extend of MC accumulation in LNs.

### LN Microenvironment and Activation of MCs

Although MC accumulation in LNs, degranulation, and their distribution in LN compartments have been frequently reported in a variety of human and mouse pathologies, our knowledge regarding the mechanism(s) of activation is still rudimentary. Ginsburg and colleagues in their study aimed to investigate the potential of the LN microenvironment and its cells in activation of MCs. For this purpose, they injected BALB/C mice intraperitoneally with horse serum, 8 injections twice weekly, and the animals were sacrificed 10–12 days later, LNs were collected, and cell suspensions were prepared and cultured on C3H mice embryonic skin monolayers. They compared the formation of MC clones in immunized and non-immunized mice (by transferring the trypsin-treated cultured cells, fixing with formaldehyde, and adding toluidine blue) and assessed the total content and released histamine in culture supernatants. Their findings showed that (a) in LN suspension cell cultures from immunized mice, MCs were detectable on day 4 onward; (b) these MC clones consisted of 3–4 MCs, each containing a few tiny metachromatic granules visualized around Golgi; (c) MC clones also formed in cultures based on suspensions prepared from non-immunized mice, but comparing with MCs from immunized mice, these MCs were lower in number and smaller in size. (1.5–3 × 10^6^ MCs (40–60% of the total cell population) vs maximal 10^6^ MCs (10.7% of total cell population) respectively); (d) MCs developed from both types of cultures had similar histamine content (3 to 7 pg/10^6^) [74]; (e) there were significantly higher amounts of free histamine in the cultures from immunized mice, suggesting a mechanism of activating and degranulating system [75]. Although the provided data unraveled important aspects of MC proliferation, the mechanism responsible for the differences between the two studied groups was not determined. Since MCs express receptors for IgG and IgE [76–78], the research team managed to determine if the mechanism of MC activation was immunoglobulin (Ig) dependent and which class of Igs was responsible. For this aim, they added heat-inactivated antiserum collected from LN donor mice to cultures containing the antigen and observed a notable degranulation and release of histamine. This experiment revealed that the possible MC activating mechanism was IgG1 but not IgE as IgG 1 is heat-stable, but IgE is heat-labile [75].

MCs isolated from different anatomic sites of a mammalian (such as LNs) do not necessarily react to activation stimuli in a similar way. Shanahan and colleagues, to compare the responsiveness of LN-MCs to activating stimuli, infected the rats with *Nippostrongylus brasiliensis* and collected the MCs from tissue culture from the mesenteric LNs (MC_MLN_), MCs isolated from intestinal mucosal (MC_IM_), and peritoneal mast cells (pMCs). Their results obtained from the comparison of these three MC types showed functional and content differences in which MC_MLN_ and MC_IM_ had comparable histamine and rat MC protease type II (or rMCP-2, belonging to β-chymase subfamily) content, while pMCs lack rMCP-2, all MC types were able to release histamine. MC_MLN_ and MC_IM_ showed low responsiveness to calcium ionophores and were unresponsive to C48/80 (depending on the extent of MRGPRX2 expression) and bee venom peptide 401 (a highly basic, 22 amino acid residue component of bee venom capable of degranulating MCs [79]); the activation of MC_MLN_ and MC_IM_ unlike pMCs could not be suppressed by disodium cromoglycate. As for morphology, MC_IM_ were smaller (8–12 μm) than pMCs (17–19 μm) and had less granule density. MC_MLN_ and MC_IM_ were similar in size, shape, and granule density [80].

The primary MC activation mechanism is IgE: FcεRI [81] and B cells and plasma cells that produce antigen-specific IgE are arranged in compartments where the presence of MCs has been previously documented. Therefore, IgE is considered to have a role in the activation of LN-MCs. To investigate the production and biofunction of IgE and anti-IgE-binding cells in LN, Mayrhofer et al. infected the Wistar rats with *Nippostrongylus brasiliensis* and then collected the LNs and stained IgE. They reported that the major site of IgE production (plasma cells) was in the mesenteric LNs (regional LN of the small intestine). Moreover, plasma cells were also found in the axillary LNs. Terminally differentiated MCs (Alcian blue positive/anti-IgE binding cells) were abundant in the capsules and hilar regions of LNs [82].

## Mast Cell-Released Mediators in the Orchestration of Immune Responses in Lymph Nodes

### MC Mediators and LN Hypertrophy

It should be considered that both LN-resident MCs and tissue MCs may influence the orchestration of immune responses in LNs. The second-mentioned MC population remotely uses mediators released from activated MCs to exert their biofunctions on LN structure and function. To study these interactions, McLachlan et al. used a mouse model of bacterial-mediated local infection by injecting bacteria into the footpads of WT and MC-deficient mice WBB6F1-W/W^v^ (W/W^v^) mice and monitored the hypertrophy response in local LNs, which was more pronounced and significant in WT mice compared to W/W^v^ mice. The next step confirming the direct involvement of MCs in this process was the specific activation of MCs in WT mice using C48/80. The finding of this test also showed a large population of activated MCs and the swelling of LNs. Additionally, they used a reconstitution strategy to restore the MC population in W/W^v^ mice by injection of MCs before bacterial challenge, which could restore the response comparable to WT mice.

Further investigation of MC mediator levels in LNs showed that MC-released TNF is markedly increased in LNs, which may contribute to the recruitment of immune cells. (T cell population increased by three-fold post-MC activation). Finally, tryptase and histamine were not involved in nodal hypertrophy [83].

The remote-controlling effect of MCs on the hypertrophy of LN can be TNF-independent. Peptidoglycan is capable of activating innate immune cells once recognized by PRRs mainly TLR2 [84], nucleotide-binding oligomerization domain (NOD)1, NOD2, and peptidoglycan recognition proteins [84, 85]. Additionally, it can activate the complement system [86]. A group of researchers sought to investigate LN hypertrophy and Langerhans cell migration in response to *S. aureus*-derived peptidoglycan. Injection of peptidoglycan into ear pinna of W/W^v^ and WT mice and the cellularity of the auricular LNs was studied after 18 h. The results obtained from WT mice showed that in LN draining the peptidoglycan-injected site, the total cells (of which CD3-positive T cells were the prominent increased cell type) were substantially increased compared to the LN draining the saline-injected site on the other ear. This effect was not observed in the W/W^v^ mice, suggesting a role for MCs in induction of the hypertrophy. In this study, MC-reconstituted W/W^v^ mice could restore the hypertrophic response. Moreover, Langerin (CD207) positive cells (Langerhans cells) were notably higher in the LNs of the ear injected with peptidoglycan in WT mice when compared to the local LN of the other ear injected with saline. In a similar pattern to the previous experiment, no significant increase was observed in the number of the Langerhans cells in the local LNs of W/W^v^ mice injected either with peptidoglycan or saline.

They also used TNF-deficient and control mice and applied the same methodology and found that both groups had a comparable increase in LN cellularity, suggesting no significant TNF-dependent mechanism involved. They completed another step by injecting purified mouse anti-trinitrophenyl (TNP) IgE, and after 2 weeks, TNP was injected to check the IgE-mediated activation of MCs in TNF-deficient mice. An increased cellularity was observed in local LN of WT mice with TNP-injection, whereas this effect was abrogated in TNF-deficient mice.

They concluded that peptidoglycan-induced migration of Langerhans cells is partially TNF independent, while IgE/Ag-induced migration of Langerhans cells is TNF dependent.

They also used C3-deficient mice, used again the same methodology, and reported that, unlike control mice, C3^−/−^ mice did not show a significant increase in the number of Langerhans cells in the local LN in ear injected with peptidoglycan compared to the saline-injected ear [87].

### Insect-Bite-Dependent Activation of MCs and Orchestration of Immune Responses in LNs

The insects’ secreting fluids later being injected into their hosts including venoms or saliva have been frequently reported to induce immune responses due to the chemical properties and high immunogenicity of the contents [79, 88]. Considering that MCs strategically are found with high density mostly in derm where insect bite penetrates, Demeure et al. in their mouse model studied the role of MCs in the trafficking of cells into local LN and the related immune responses in mice once the animal was exposed to *Anopheles* mosquitos. To this aim, they injected WBB6F1- ^+/+^ (WT) and WBB6F1-Kit^W^/Kit^W−v^ (W/W^v^) mice with Evans blue (to visualize vascular permeability) and then exposed to mosquito bites for 1 h. The blue stains around hemorrhagic bites were only seen in WT mice, suggesting a role for MCs. Furthermore, restoration of the MC population in W/W^v^ mice and then repeating the experiment could restore the effect. They checked the direct effect of MC presence in LN hypertrophy and cell recruitment. In mice with functional MCs (WT and MC-restored W/W^v^ mice), leukocyte density and CD11c + cells were significantly increased compared to the W/W^v^ mice. Interestingly, the application of toluidine blue and chloroacetate esterase staining showed the degranulation of MCs (up to 30%) surrounding the biting site. Collecting of the inguinal LNs in mice exposed to *Anopheles* mosquitos showed significant hypertrophy. Analysis of the cellularity of LNs was performed using flow cytometry, and CD11c + DC, CD11b + cells (macrophages), CD3 + (T cells), and B220 + (B cells) recruitment was observed. Finally, they found elevated levels of TNF in LNs but failed to find a link between TNF and cell influx to LNs after using anti-TNF Ab [89].

## Mast Cells and Lymphangiogenesis

The process of lymphangiogenesis, in which pre-existing lymphatics give rise to new lymphatic vessels, happens through a variety of physiological and pathological reactions. It has a pivotal role in metastasis and inflammation by accelerating immune cell trafficking [90, 91].

MCs have been linked to the extension of the lymphatic network largely via releasing mediators including VEGF-C and -D. Recently, Cho and colleagues designed a study based on utilizing BALB/c, C57BL/6 J, and MC-deficient cKit^w−sh^ mice and the application of MC stabilizers to check the role of MCs in lymphangiogenesis at the ocular surface. In this model, a single suture was intrastromal placed on the cornea and in a group of mice, sodium cromolyn in PBS, and in the other group as control only PBS was topically administered. This treatment was frequently repeated before and after suture placement until 1 week, and then their corneas were collected to prepare a single-cell suspension.

Furthermore, they used human primary lymphatic endothelial cells (LECs) and cultured them with a variety of endothelial growth factors, including VEGF, FGF, EGF, and IGF, or cocultured them with LAD2 cells. LECs were then cultured on a gel matrix to monitor the tube formation.

Below, we listed their main findings: (a) Fluorochrome-conjugated-avidin stained corneas from naïve and suture-placed Balb/c mice showed MCs in proximity to the peripheral lymph vessels; (b) new lymph vessels were developing alongside infiltrated cKit^+^ FCεR1^+^ MCs; (c) mRNA expression of VEGF-D was significantly increased in corneas with suture placement; (d) in their in vitro experiment, no tube formation was observed in LECs cultured in basal media alone; however, those co-cultured with LAD-2 exhibited a substantially higher tube formation activity; (e) LECs co-cultured with LAD-2 cells showed a proliferation rate comparable to LECs cultured with growth factors (both significantly higher than those cultured alone); (f) MCs produced VEGF-D by which they induced the proliferation and development of LECs; (g) tear wash samples collected 6 h post-suture placement from cKit^w−sh^ and control mice (C57BL/6) were analyzed for tryptase. It showed considerable levels of tryptase only in control mice exhibiting activated MCs; (h) cKit^w−sh^ mice unlike the WT mice showed no significant change in VEGF-D expression; (i) cromolyn-treated corneas had lower expression levels of VEGF-D when compared to PBS-treated corneas [92].

## Mast Cells and Extension of LN Vasculature

Furthermore, MCs have been known to play a role in angiogenesis in different settings by releasing proangiogenic mediators such as IL-8, TNF, VEGF, bFGF, and TGF-β [93–96]. The role of MCs in the extension of LN vasculature was an interesting subject Ribatti et al. focused on to find a link between MCs and the endothelial network of LNs. They used a number of sentinel LNs from patients with breast cancer and divided them into two groups (each containing 40 patients) with and without micrometastases and applied anti-CD31 and tryptase to locate endothelial cells and MC, respectively. They found that the extent of angiogenesis was correlated with the count of tryptase + MCs and MCD was significantly higher in LNs with micrometastases compared with those without (*P* < 0.001) [97].

The proangiogenic role of tryptase acting through endothelial-expressed protease-activated receptor-2 (PAR-2) in LN microvascular density (MVD) was studied in gastric cancer. The main finding was the presence of a significant correlation between tryptase + MCs, and MVD was found in primary tumors and metastatic LNs [98].

The positive correlation of MCD with tumor-microvessel density (TMD) in dogs highlighted the role of MCs in LN vasculature extension. In one study, nodal lymphomas collected from 33 dogs were compared with non-neoplastic LNs of controls by staining MCs and vasculature using toluidine blue and Factor VIII, respectively. The results showed that MCD in lymphoma was significantly higher than controls and that MCD was correlated with TMD. Increased vascularization and MCs were observed on the invasive edge (periphery) of the neoplasm [99].

## MCs in Hodgkin Lymphoma

The cytologic characterization of Hodgkin’s disease is the presence of a few malignant cells, the mononuclear proliferating Hodgkin cells (20–30 μm in diameter), and the multinuclear Reed-Sternberg (HRS) cells (up to 100 μm in diameter) in the lymphomatous tissue [100, 101]. Although Hodgkin and HRS cells are derived from mature B cells, however, instead of having a B cell phenotype, they are found with an exceptional co-expression of markers of different hematopoietic cells [102].

Additionally, HRS cells have an altered profile of cytokines (including chemokines) recruiting other cell types into the LNs. For instance, CCL17 (also known as thymus and activation-regulated chemokine (TARC)) and CCL11 (eotaxin) released from HRS cells play a role in chemoattraction of T cells (expressing CCR4) [103] and eosinophils [104] into LNs, respectively. Three interconnected unmet questions were: “Do MCs accumulate as infiltrating cells in HD-affected LNs? “, if so, “Have HRS cells a role in this process?”, and finally “What is the responsible cytokine(s)?”.

It was already known that MCD increases in HD-affected LNs. In line with this, Crocker et al. reported a high MCD in the LNs of their studied patients showing follicular hyperplasia. They found MCs in particular close to the sinuses and the interfollicular regions. Also, MCD was elevated in specimens showing nodular sclerosis [105]. Moreover, the higher MCD, clinically, gained significance when it was reported that MCD correlates with a poorer diagnosis [106]. Indeed, the outcome of tumor infiltration of MCs is a tumor-specific feature, and MC infiltration may be linked to a better or worse prognosis according to the tumor type and the involved organ (to have a more comprehensive vision of this concept, please refer to Table 1 in the following reference [107]). Fischer and colleagues studied the chemokine network shaped surrounding HRS cells to find the chemokine responsible for MC recruitment. For this aim, they used cord blood MCs (CBMCs) and a number of HD cell lines including CO, DEV, HDLM-2, KM-H2, and L540 as well as tissue sections from HD-affected LNs, and their RNA was extracted. Then, they analyzed the expression levels of chemokines in the studied cell lines. To investigate the chemotactic capacity of CCL5 on CBMCs, the team performed a chemotaxis assay using a Boyden chamber consisting of nitrocellulose filters (pore size = 8 μm) coated with fibronectin. They placed CBMCs in the upper and conditioned medium collected from HDLM-2 in the lower chamber and used recombinant CCL5 (rCCL5) as the positive control. The results showed that (a) the cell lines had each a completely different profile of chemokine expression; (b) HDLM-2, L540, and KM-H2 expressed CCL5 mRNA (DEV cell line expressed it weakly); (c) ELISA results showed that HDLM-2, L540, and KM-H2 release CCL5 protein; (d) IHC findings showed that HRS cells in LNs produce CCL5; (e) conditioned medium induced CBMCs’ migration similar to rCCL5; (f) application of anti-CCL5 MAb could inhibit the effect [108].

Molin et al. studied the direct cell-to-cell interaction of MCs with HRS cells. They used tumor samples from 42 patients with Hodgkin’s disease and stained them for CD30L and tryptase. Over half of the tryptase + MCs in the samples expressed CD30L, and these MCs constituted 2/3 of the CD30L-positive cells. They also checked the CD30L expression of HMC-1 and cord blood cultured human MCs developed in vitro and confirmed their expression of CD30L. The significance of CD30L on MCs is due to its biofunction to induce DNA synthesis (thus proliferation) in CD30-positive cells. To determine this aspect, they cocultured CD30L positive-HMC-1 with several HD cell lines including HDLM-2, DEV, KM-H2, and L540, fixed them using paraformaldehyde, and checked the DNA synthesis. Their findings confirmed that CD30L-CD30 axis activation induces DNA synthesis in the responder cells by checking their uptake of [3H]- thymidine. Additionally, the application of anti-CD30L mAb could reverse the effect and confirm the involvement of the CD30L-CD30 axis [101].

Further investigation of MC-HRS interaction in nodular sclerosis subtype of classical Hodgkin lymphoma (NSCHL) and non-NSCHL showed that MCD was significantly higher in NSCHL (1.69 ± 0.37/mm^2^) than in non-NSCHL (0.24 ± 0.09/mm^2^), (*P* = 0.0001). There was a positive correlation between MCD and fibrosis (correlation coefficient: 0.852, *P* < 0.0001). MCs are likely to infiltrate LNs by IL-13-producing HRS and release IL-13 and TGF-β that promote fibrosis in NSCHL [109].

## Involvement of Lymph Nodes in Mastocytosis and Systemic Mast Cell Disease (SMCD)

Mastocytosis is defined by excessive proliferation and accumulation of abnormal MCs in terms of morphology and phenotype in one or more organs and tissues including the skin, bone marrow, spleen, liver, and LNs [110]. Horny et al. investigated the LNs of patients with mastocytosis and focused on infiltrated LN-MCs. In their 21 studied LNs diagnosed with SM, most LNs (80%) had MC infiltrates commonly visualized in the medullary cords and sinuses. Moreover, several histological alterations were observed, which possibly were linked to the biofunction of MC-released mediators. These alterations included GC hyperplasia, vessel hyperplasia, eosinophilia, plasmacytosis, and collagen fibrosis. Two distinct patterns of MC infiltration including focal and diffuse were observed. The latter was the dominant pattern. Finally, of 17 cases with MC infiltration, only 4 were intact in terms of architecture, while 10 were found with partial destruction and three with thorough destruction [111].

Systemic mastocytosis (SM) is an uncommon illness characterized by the multiorgan accumulation of clonal MCs that differentiate less dependently on the natural KIT ligand (SCF) because of somatic mutations in c-KIT (D816V mutation presents in over 90% of adult patients). Depending on the organs affected and mediators released, the induced symptoms may vary including diarrhea, flushing, itching, and others [112–115]. LNs may be affected in SM; however, we still do not fully understand the structural alterations and prognosis, and what we currently know is based on case reports. For instance, in one case, the enlargement of retroperitoneal LN was reported in SM [116].

An investigation of 23 LN samples collected from 19 individuals with SMCD revealed that MC infiltrates were present in various anatomical sites of LNs with different frequencies of involvement in which paracortex (88%), parafollicular region (50%), follicles (25%), medullary cords (13%), and the sinuses (6%) were affected. MCs were abundant commonly in perivascular regions [117].

## Clinical Implications of Mast Cells In Immunologic Homeostasis of Lymph Nodes

The association of MCD with the progression of tumor depends on the type of tumor. MC infiltration in breast tumors and involvement of local LNs and prognosis have been studied. In a study, 104 cases of invasive breast carcinoma were studied for tumor and LN-MCD, involvement of LN, and metastasis, and it was tried to find a link between MCD with prognosis. The findings showed that parallel to the increase in tumor size and volume increase, MCD increased comparably in metastatic LNs. Furthermore, several other features including lymphovascular, lymphatic, and perineural invasion correlated with intratumoral MCD. The MCD reported in metastatic LNs and tumors showed a significant correlation [118]. The correlation between LN-MCD and survival time in breast cancer has been reported in which those patients with survival time > 60 months had > 11 MC/mm^2^ [119].

Investigation of the positivity of MC-specific proteases in LNs with or without metastasis in humans revealed that tryptase, a MC-specific protease crucial to angiogenesis [120] (thus progression of metastasis), may be used as a marker to predict the prognosis of patients before radical surgery. In a study performed on LNs of patients with gastrointestinal cancer, MC and tryptase staining revealed that the number of MC positive to tryptase and the number of metastatic LNs was correlated (*r* = 0.88; *P* = 0.01). Additionally, the number of tryptase-positive MCs in tumor tissue and the number of nonmetastatic LNs were inversely correlated (*r* = −0.74; *P* = 0.03) [121].

Another important clinical implication of MCs in LN metastasis could be the expression of different preformed mediators (mainly proteases) and possible application in grading the tumors. Similar work has been done in canine models of MC tumors (MCTs) by investigating the mRNA expression levels of CPA3 and tryptase with the grade of metastasis of MCTs (HN0-HN3, where HN3 implies overt metastasis). Additionally, the expression levels of tryptase and CPA3 were substantially upregulated in LNs with HN2 and HN3 grades. Profiling mRNA or protein levels of MC mediators in human LNs surgically removed for pathological diagnosis may provide further biomarkers in grading the tumor and tracking the metastasis [122].

Tumor-associated antigen (TSA)-pulsed DCs gained attention in immunotherapy of cancer. One measure of a therapeutic vaccine’s effectiveness is the rate at which adoptively transplanted DCs mobilize to LNs. Tissue MCs are already known for their capacity to drive and mobilize DCs toward LNs once they become activated. In a study, activation of C57BL/6 mice skin MCs using C48/80 speeded up the HBsAg-pulsed DCs homing to LNs and induced lymphocyte proliferation when compared to the control group with intact MCs (without C48/80 treatment) [123]. Considering the activation of MCs and more importantly, determining the involved MC mediators and using them to increase the mobilization of DCs to LN could be a promising stem in increasing the efficiency of vaccination, especially in oncology.

## Piled Up Unsolved Inquiries Regarding the Role of Mast Cells in Lymph Node Immunology

In previous sections, we reviewed different immunologic aspects of the LN-residing MCs and their participation in immune responses in detail. We also checked the limitations of studies and the way the current knowledge can be extended using a variety of methods and techniques and provided Table 2 to represent the unmet questions waiting for further research.

**Table 2:** Unmet question and corresponding literature review on MC-orchestrated immune responses in LNs

Unmet question and corresponding literature review	Ref
MC homing, location, and accumulation in LNs	
**Is the higher number of MCs in pathological conditions the result of their recruitment or local proliferation?** MCs are normally found in low numbers in LNs, but their numbers substantially increase in pathological conditions. This concept can be either due to the increase in their recruitment or local proliferation (or a combination of both). Investigation of this concept could provide interesting facts regarding the biology of MCs in LNs	[59]
**Do MCs enter LNs only via lymphatic vessels?** The current body of literature supports that MCs enter LNs via lymphatic vessels in normal conditions. However, the possibility of migration or entering through circulation, especially in the case of mastocytosis where there is a huge load of organ-infiltrated neoplastic MCs or in HD where the release of chemokines with a role in cell trafficking and integrins may be dysregulated can be a research topic	[124–126]
**Do hormonal changes alter the LN-MCD?** It was previously reported that in female BALB/c mice, uterus draining and popliteal LNs had different MCD between estrous and diestrous in which MCD in estrous animals was significantly higher. Moreover, a comparison of young and mature animals showed that as for popliteal LNs-MCD, mature animals either in estrous or diestrous had higher MCD compared to weanlings. We have a knowledge gap in understanding the effects of age and hormonal balance (and imbalance) and their alternating effects on LN-MCD in humans especially for uterus LNs. In addition to sexual hormones, other hormones such as leptin (one of the adipokines secreted from adipose tissue) are reported to have a regulatory role in LN-infiltrating MCs in mice. Indeed, leptin deficiency induces obesity and accumulation of MCs in retroperitoneal, mesenteric, and inguinal LNs of leptin-deficient mice	[127–129]
MCs interaction with other immune cells in LN	
**Do MCs overexpress their CD30L once their various activating receptors are engaged?** MC-CD30L: CD30-HRS axis has been reported to have a role in the activation of HRS in Hodgkin’s disease. Once Milon et al. checked MCs expression of CD-30L, they found out that application of ionomycin (a calcium ionophore and stimulator of MCs) induced CD-30L expression in murine mast cell line MCP5/l which was negative before stimulation. They also found that IgE activation does not induce the marker’s mRNA expression in the same cell line. Investigation of CD-30L expression of human organ-derived MCs and other cell lines activated with receptors addressed in the introduction section could be an interesting theme of research in oncology and possibly find a link between allergy and Hodgkin’s disease and the outcome of the presence of allergy in patients with Hodgkin’s disease	[101]
**Do MCs have a role in the egression of lymphocytes from LNs?** The presence and accumulation of MCs in LN have been reported to correlate with a poor prognosis in patients with lymphomas. The impairment of lymphocyte normal retention in LNs provides the opportunity to encounter their specific antigens (matching with their expressed receptors), this process is dependent on the S1P: S1PR1 axis and CD69 expression. It would be interesting to investigate the role of MC-released mediators in altering the expression of CD69 and S1PR1 on lymphocytes. To this aim, the egression of labeled lymphocytes in MC-deficient mice and comparing the results after restoring MC populations (by reconstitution of MCs from other WT mice) and alternatively activating MCs cocultured with lymphocytes and studying the levels of CD69 and S1PR1 expression could be an interesting theme of research	[130, 131]
**Is there a MC-FRC interaction resulting in hypertrophy of LNs?** MC-produced TNF has been reported to mediate the LN swelling when MCs are activated locally outside LNs. The mechanism involved in this process is poorly understood. Since hypertrophy of an LN is directly controlled by the expansion of FRCs and their produced ECM components, there may be a remotely conducted interaction between MCs and FRCs	[83]
**Do MCs have a role in the recruitment of immune cells to LNs?** Recruitment of cells of innate immunity acting as APCs (in particular DCs) to LNs accounts for a step in the orchestration of immune responses shifting from innate immunity to adaptive immunity. DCs uptake antigens, process and present the fragments, and activate T cells. MCs have been reported to have a role in DCs recruitment into LNs in mouse models. In a study, OVA-Alexa Fluor 647 was used to immunize and track (MHC II + CD11c + B220–) mDCs from lung to LNs both in WT mice and C57BL/6-Kit^W−sh/W−sh^. Comparing the results showed that MCs actively regulate the number of DCs recruited in LNs after antigen challenge (LN-DC density in two studied mice: WT mice > C57BL/6-Kit^W−sh/W−sh^). The molecular mechanism of this process and whether MCs residing in tissue or LNs mediate it is still poorly understood	[132]
**Do MCs have a role in the dilatation of HEVs by which regulate HEV permeability?** Distribution of MCs around HEVs in rat LNs showed that HEVs surrounded by abundant MCs are unusually dilated. Investigation of this observation in human LNs has not yet been studied in detail and possible MC mediators responsible for the effect have not been investigated	[133]
MC activation in LNs	
**Do HRS cells activate MCs in the involved LNs?** HRS cells phenotypically are positive for CD30, and CD15, but negative for CD3, CD20, and CD45 and have a cytokine secretome profile including IL-5, IL-6, IL-7, IL-9, IL-10, IL-13, GM-CSF, TGF-β, and lymphotoxin α. We currently do not know if HRS cells produce MC activating mediators (such as SCF) or if there is a positive loop between MCs and HRS cells in which MCs induce HRS cells via MC-CD30L: CD30-HRS axis to produce cytokines that activate MCs. Another level of interaction between MC and HRS may exist due to the responsiveness of HRS cells to MC cytokines by expressing IL-6R and IL-13R	[134–138]
**Does MRGPRX2 have a role in the activation of LN-MCs?** MRGPRX2 is a highly expressed activating receptor on skin MCs and these MCs were mentioned in the above sections to migrate from skin into LNs. LNs, like other human tissues and organs, are rich in neurons such as sensory neurons which release neurotransmitters including SP, VIP, etc. We think that it deserves particular attention to investigate whether MRGPRX2 has a role in the regulation of MC function and the extent of their activation by receiving activating signals from neural networks	[139–142]
MCs interaction with endothelial and lymphatic vessels	
**Is there a link between LN-MCD and the increase in the number of HEVs after antigen challenge or microbial exposure?** The vasculature network of LN dramatically extends and the size and number of HEV increase in LNs to support extravasation of further numbers of lymphocytes into their specific compartments in LN. Additionally, the expression profile of molecules on HEVs responsible for this process undergoes extensive changes (such as an increase in the expression of P- and E-selectins, and CXCL9 chemokine) MCs have also been reported to accumulate surrounding HEVs. They likely have a role in HEVs network extension and the expression of surface molecules and its chemokine network. Comparison between WT and MC-deficient mice or WT mice receiving MC stabilizers can be an effective approach to evaluate these changes	[71, 143]
**How does TNF mediate LN hypertrophy when it reaches LN via afferent lymphatics?** The current rationale supports the idea that TNF released from MCs in peripheral tissues is drained in lymph and reaches the LN via afferent lymphatics, however, it yet remains unclear how TNF exerts such biofunction as it is released in low concentrations, then becomes diluted in lymph and more importantly it has a fairly low half-life	[144]
Clinical implications of MCs, their mediators levels in LNs in a variety of pathologies	
**Can a panel of MC-released mediators be developed to predict the involvement of LNs and tumor metastasis?** Our understanding of MC involvement, activation, and most importantly the effects of its mediators on LN involvement (as a critical feature of metastasis) is imperfect and limited. It would be interesting to compare the levels of MC-mediators in patients with different types of tumors either metastatic or not to suggest a possible panel to predict the metastasis	[116]
**Does a higher MCD in tumor-free LNs have a clinical significance in the progression or prognosis of disease?** A higher MCD in tumor-free mediastinal LNs compared to metastatic nodes collected from lung cancer patients was reported. The outcome of their accumulation has not been investigated in different tumors. The presence of a higher number of MCs may facilitate metastasis as the tumors spread. Additionally, MCs produce several proinflammatory cytokines, and their release in the tumor microenvironment (TME) establishes the inflammation. The inflammatory response within the TME may act in favor of tumor progression	[59, 107]
**Can MC location in LN and its vicinity to anatomical structures of LN be clinically considered as a diagnostic feature?** MCs have been reported in the T cell zone of tumor-free LNs (while B follicles were almost free of MCs) Their location within the anatomic structure of an LN may be linked to the prognosis, however, their accumulation sites may differ in different pathologies (Table 1)	[59]

## Discussion and Conclusion

LNs, because of their unique structure and function, play a crucial role in the orchestration of immune responses.

Each LN acts as a trap to maximize the contact between invaders (gathered from tissues and directed into lymph circulation) and professional APCs to be presented to lymphocytes with matching receptors. The efficient blood circulation carrying naïve lymphocytes into LN guarantees the facing of lymphocytes with recognition receptors matching the presented antigen [5, 145].

For this aim, naïve lymphocytes enter the LN from the blood via HEVs anatomically located within the T-cell zones. They continuously migrate through the T-cell regions to the follicle. Once lymphocytes encounter their presented specific antigen become activated, and remain in the same compartment, proliferate, and differentiate (the total process is called “clonal expansion”). Otherwise, they find their way out of LN through the efferent lymphatic vessel, which finally redirects unmatching lymphocytes to the blood to start entering another LN probably with other presented antigens.

At a molecular level, the egression of lymphocytes from LNs depends on the sphingosine-1-phosphate (S1P) gradient (higher levels in blood and lymph than tissues), its binding to S1PR1 expressed on lymphocytes, and CD69 expression. The latter is considered an early marker of lymphocyte activation. Once a lymphocyte becomes activated, CD69 impairs the expression of S1PR1, resulting in retention of the lymphocyte within the LNs [130, 146, 147]. This steadily continued monitoring of lymph-carrying antigens helps the mammalians to use their rich repertoire of lymphocytes each capable of antigen recognizing and in return proliferating and generating a unique antigen-specific clone to respond to the antigen [145, 148].

MCs presence in LNs is a common finding not only in humans but also in a variety of other mammals such as mice, opossums, and dogs [149]. MCs are elaborately located in different LN compartments of LNs in mammals based on the type and anatomic location of LNs which is necessary to their direct interplay with other immune and stromal cells of LNs. Therefore, MC distribution in LN is not only unique in different species but even among different LNs of the same species (or individual). For instance, in opossums, para-aortic, common iliac, cardial pyloric, and cecocolic have high lymphatic sinus MCs (LSMC), while these cells are rarely reported in the mesenteric, pyloric, and head-of-pancreas LNs [150]. Such variations in the type and location of LN-MCs make the understanding of their function and crosstalk even more complicated. On the other hand, the infiltration of MCs into different compartments of LN depends on the metastatic or non-metastatic status of LN or the type of invading microorganisms (Table 1), which makes the study of LN-MCs even more challenging. According to Table 2, there are still important aspects of LN-MCs yet to be investigated, and this list will be extended over time along with emerging new technologies or introducing new pharmaceuticals affecting MCs survival. In line with this, Lirentelimab (AK002) suppresses MCs by binding to Siglec-8, or Barzolvolimab (CDX-0159) prevents cKIT dimerization and deprives the cell of its surviving growth factor SCF [151–153]. Additionally, another group of potential pharmaceuticals can be KIT-tyrosine kinase inhibitors (KIT-TKIs) that have recently become available or are under development. Examples include imatinib [154], avapritinib [155], elenestinib [156], and bezuclastinib [156]. The impacts of these newly introduced agents on the orchestration of MC-mediated immune responses and reshaping the LN structure and function are expected to attract attention to perform more research on LN-MCs.

## Data Availability

No datasets were generated or analysed during the current study.

## Abbreviations

APCs: Antigen presenting cells
BSA: Bovine serum albumin
CBMCs: Cord blood MCs
CHS: Contact hypersensitivity
CPA3: Carboxypeptidase A3
CXCL13: CXC-chemokine ligand 13
DNFB: Dinitrofluorobenzene
fDCs: Follicular dendritic cells
HRS: Reed/Sternberg
FRCs: Fibroblastic reticular cells
GCs: Germinal centers
GFP: Green Fluorescent Protein
HD: Hodgkin's disease
HEVs: High endothelial venules
HRP: Horseradish peroxidase
iDCs: Interdigitating dendritic cells
IgE: Immunoglubolin E
JP-8: Jet propulsion-8
LECs: Lymphatic endothelial cells
LN-MCD: Lymph node MC density
LT: Leukotriene
LVD: Lymphatic vessel density
MCps: MC progenitors
MCs: Mast cells
MHC-II: MHC class II molecule
MIP-1: Macrophage inflammatory protein-1
MMP-9: Matrix metalloproteinase-9
MPO: Myeloperoxidase
MRGPRX2: Mas-related G protein-coupled receptor X2
MVD: Microvascular density
NALT: Nasal-associated lymphoid tissue
NOD-1: Nucleotide-binding oligomerization domain-1
NSCHL: Nodular sclerosis subtype of classical Hodgkin lymphoma
PAFR: Platelet-Activating Factor Receptor
PAR-2: Protease-activated receptor-2
pDCs: Plasmacytoid dendritic cells
PD-L1: Programmed Death-Ligand 1
PG: Prostaglandin
PMC: Peritoneal mast cells
PPs: Peyer's patches
PRRs: Pattern recognition receptors
RANKL: Receptor activator of nuclear factor κB ligand
rMCP-2: Rat MC protease type II
SCF: Stem cell factor
SHM: Somatic hypermutation
SM: Systemic mastocytosis
SP: Substance P
TARC: Thymus and activation-regulated chemokine
TBM: Tingible body macrophage
TILs: Tumor-infiltrating lymphocytes
TMD: Tumor-microvessel density
TME: Tumor microenvironment
TNF: Tumor necrosis factor
TNP: Trinitrophenyl
TSA: Tumor-associated antigen
UV: Ultraviolet radiation
VIP: Vasoactive intestinal peptide
WT: Wild type
